# Linarin Inhibits the Acetylcholinesterase Activity *In-vitro *and *Ex-vivo*

**Published:** 2015

**Authors:** Xinchi Feng, Xin Wang, Youping Liu, Xin Di

**Affiliations:** School of Pharmacy, Shenyang Pharmaceutical University, 103 Wenhua Road, Shenyang 110016, PR China.

**Keywords:** Linarin, Acetylcholinesterase inhibitor, Molecular docking study, Mice brain

## Abstract

Linarin is a flavone glycoside in the plants *Flos chrysanthemi indici*, *Buddleja officinalis*, *Cirsium setosum*, *Mentha arvensis* and *Buddleja davidii*, and has been reported to possess analgesic, antipyretic, anti-inflammatory and neuroprotective activities. In this paper, linarin was investigated for its AChE inhibitory potential both *in-vitro* and *ex-vivo*. Ellman’s colorimetric method was used for the determination of AChE inhibitory activity in mouse brain. *In-vitro* assays revealed that linarin inhibited AChE activity with an IC_50_ of 3.801 ± 1.149 μM. *Ex-vivo* study showed that the AChE activity was significantly reduced in both the cortex and hippocampus of mice treated intraperitoneally with various doses of linarin (35, 70 and 140 mg/Kg). The inhibition effects produced by high dose of linarin were the same as that obtained after huperzine A treatment (0.5 mg/Kg). Molecular docking study revealed that both 4’-methoxyl group and 7-*O*-sugar moiety of linarin played important roles in ligand-receptor binding and thus they are mainly responsible for AChE inhibitory activity. In view of its potent AChE inhibitory activity, linarin may be a promising therapeutic agent for the treatment of some diseases associated with AChE, such as glaucoma, myasthenia gravis, gastric motility and Alzheimer’s disease.

## Introduction

Acetylcholinesterase (AChE) is a substrate specific enzyme expressed in many tissues degrading neurotransmitter acetylcholine (ACh) in the nerve synapses. Inhibition of AChE is considered as a promising strategy for the treatment of some diseases caused by the too low level of ACh, such as glaucoma, myasthenia gravis, gastric motility and Alzheimer’s disease (1-4). Nowadays, a few synthetic AChE-inhibiting drugs are used (e.g. donepezil, rivastigmine and tacrine), however, some cholinergic adverse effects such as nausea, vomiting and diarrhoea are the major drawbacks (5). Therefore, the search for new AChE inhibitors with higher efficacy and less side effects is still of interest (6-9).

**Figure 1:**
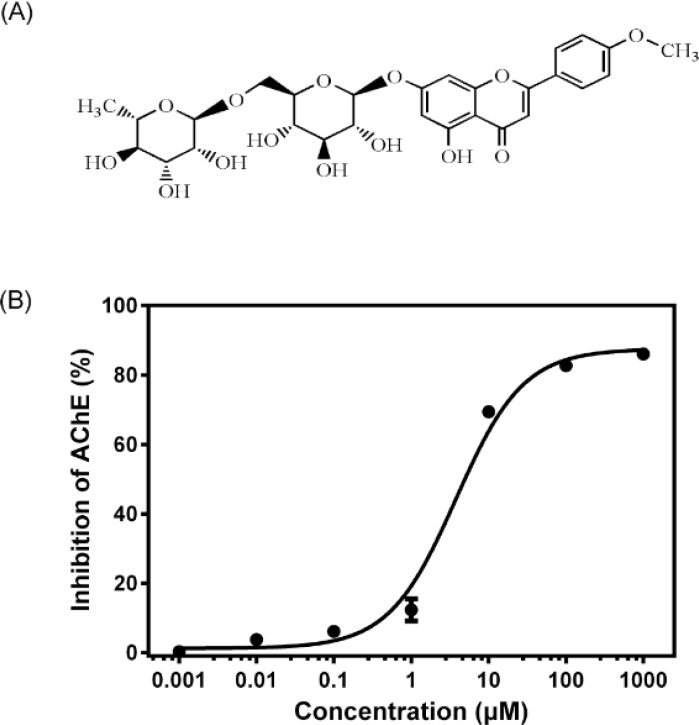
(A) Chemical structure of linarin. (B) *In-vitro* AChE inhibition curve for linarin (0.001–1000 μM). Data are expressed as mean ± SD (*n* = 3).

Linarin (acacetin-7-O-β-D-rutinoside, Figure 1 A) is a flavone glycoside present in many medical plants, including *Flos chrysanthemi indici*, *Buddleja officinalis*, *Cirsium setosum*, *Mentha arvensis* and *Buddleja davidii*. It has been reported to possess many pharmacological activities, such as analgesic, antipyretic, anti-inflammatory and neuroprotective effects (10-12). In a recent study, linarin was identified as the active component responsible for the ability to inhibit AChE activity of some crude extract of *Mentha* by a LC−MS−MS assay without further purification (13). The quantitative determination of the AChE inhibitory potency of linarin and the possible mechanism remained to be elucidated. Therefore, in this study, pure linarin was obtained and both *in-vitro* and *ex-vivo* models were used to better characterize the AChE inhibitory potency of linarin. Moreover, molecular docking simulation was also conducted to find the possible molecular mechanism. These studies will help to understand the role and mechanism of linarin in inhibiting AChE activity.

## Experimental

*Chemicals and reagents*

Linarin (>98% purity, isolated from *Cirsium setosum*) was obtained from Chengdu Must Bio-technology Co. Ltd (Chengdu, China). Huperzine A was obtained from National Institute for the Control of Pharmaceutical and Biological Products (Beijing, China). Acetylthiocholine iodide (ATCI) and 5,5'-dithiobis-(2-nitrobenzoic acid)(DTNB) were obtained from Shanghai Ruji Bio-technology Co. Ltd (Shanghai, China). Dimethyl sulfoxide (DMSO) was purchased from Tianjin Bodi Chemical Co. Ltd (Tianjin, Chian). All other reagents and organic solvents were of analytical grade.

*Animals*

Healthy male Kunming (KM) mice, weighing 20 ± 2 g, SPF, were provided by the Laboratory Animal Center of Shenyang Pharmaceutical University (Shenyang, China). All animals were housed under standard controlled environmental conditions: temperature (25 ± 2 °C), relative humidity (60 ± 10%), room air change (12–18 times/h) and light/dark cycle (12/12). Food and water were available ad libitum*. *The study was conducted under protocols approved by the Animal Ethics Committee of Shenyang Pharmaceutical University, in accordance with the Guide for the Care and Use of Laboratory Animals (NIH publication No.85-23, revised in 1985).

*In-vitro AChE inhibition assay*

After the mice were killed by decapitation, the brains were quickly removed on an ice-cold plate and homogenized in a 10-fold volume of cold 10 mM phosphate buffer (pH 7.4). The homogenates were centrifuged at 3500 rpm for 10 min and the supernatants were employed as sources of enzyme. All the above steps were carried out at 4 °C. AChE activity was determined according to a modified Ellman assay method (14). Briefly, 150 μL of the brain homogenates and 600 μL of different concentrations (0.001–1000 μM) of linarin (dissolved in DMSO and then diluted in 10 mM phosphate buffer with final DMSO concentration less than 1%) were mixed. After the mixture was pre-incubated at37°C for 5 min, 2100 μL of 1 mM DTNB and 300 μL of 3 mM ATCI in 10 mM phosphate buffer (pH 7.4) were added to start the reaction. The increase in absorbance due to the formation of 5-thio-2-nitrobenzoate anion was measured during 1 min at 405 nm and 37 °C. Huperzine A at a concentration of 150 μM was used as a positive control. The AChE activity was estimated through changes of absorbance per minute and the results were reported as percentage of inhibition to AChE activity, where the optical density measured from blank (without inhibitor) was considered to be 0% of inhibition and the optical density measured from control (huperzine A) was considered to be 100%. The percentage of inhibition was calculated as follows: (*A *_blank_ − *A *_sample_)/( *A *_blank_ − *A *_control_)× 100%, where *A *_blank_,* A *_sample_ and *A *_control_ are the absorbance of blank, sample and positive control, respectively. The half maximal inhibitory concentration (IC_50_) was calculated from the Prism (GraphPad 6.0, Dose-response - Inhibition) curve obtained by plotting the percentage inhibition versus the concentration of linarin.

*Ex-vivo AChE inhibition assay*

The mice were randomly divided into five groups of six mice each: normal control group, huperzine A (0.5 mg/Kg) treated group and low-, moderate-, high-dose linarin(35, 70 and 140 mg/Kg) treated groups. The mice in the normal control group received an equivalent volume of 5% DMSO in saline (vehicle). All the mice were treated with a single intraperitoneal injection of the respective drugs and sacrificed 30 min later. The brains were quickly removed on an ice-cold plate, and then the cortex and hippocampi were dissected out. These tissues were homogenized in a 10-fold volume of cold 10 mM phosphate buffer (pH 7.4). The homogenates were centrifuged at 3500 rpm for 10 min and the supernatants were employed as sources of enzyme in AChE assay as described above. The percentage of inhibition was calculated by comparison with the AChE activity of mice treated with vehicle.

*Statistical analyses*

Statistical analyses were performed by using one-way analysis of variance (ANOVA) followed by Duncan’s multiple range test using the Statistical Package for the Social Sciences (SPSS) software.

*Molecular docking simulation*

Molecular docking simulation was performed using AutoDock 4.2. The X-ray crystal structure of acetylcholinesterase was obtained from RCSB protein data bank, which was prepared by removing both non-polar hydrogens and water molecules, and adding Kollman united-atom partial charges and solvation parameters. Meanwhile, the torsion angles of linarin were identified. A grid box of 70×70×70 points in the *x*, *y *and *z *directions were constructed with a grid spacing of 0.375 Å at the center of the catalytic pocket of acetylcholinesterase. The Lamarckian Genetic Algorithm method was used for the global search for optimum binding position. After completion of docking and clustering analysis, the most favorable binding conformation with the lowest free energies was selected as the binding pose.

## Results

**Figure 2:**
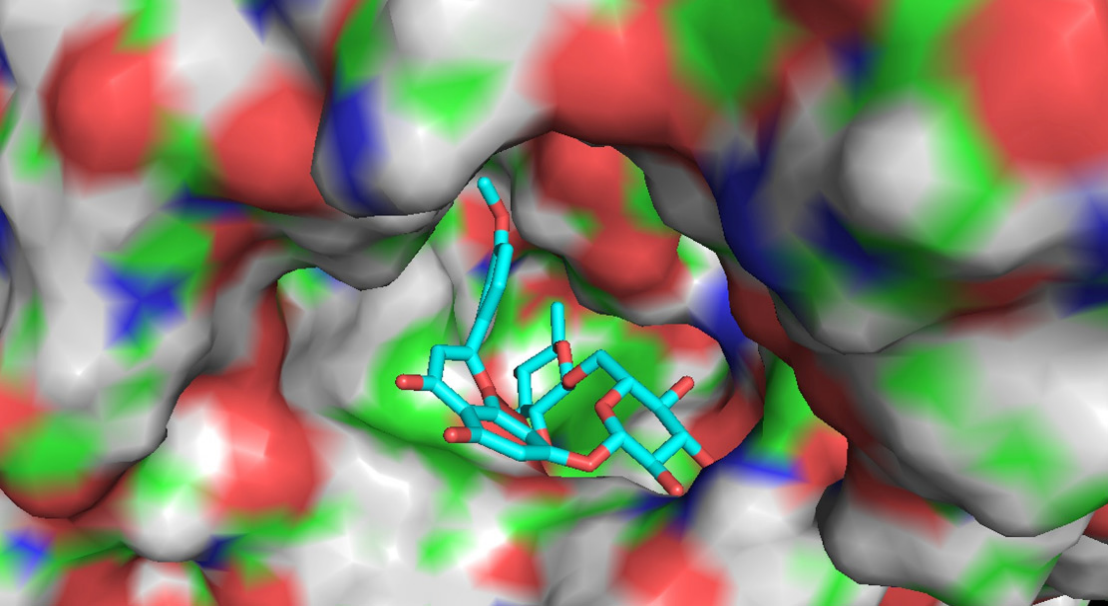
Binding mode of linarin inside active site of AChE. Linarin is shown in turquoise blue.

**Figure 3:**
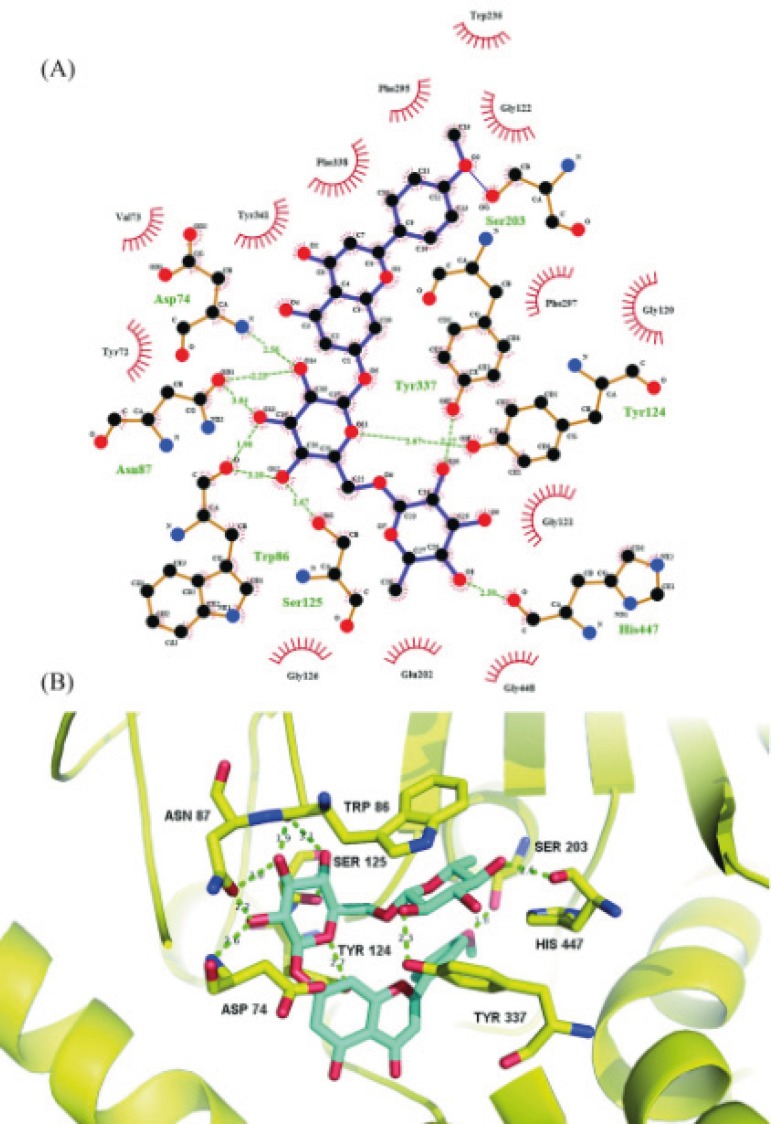
(A) Schematic illustration of the interactions of linarin with AChE. Green dotted line represents hydrogen bonds. Black: carbon; red: oxygen; blue: nitrogen. : Ligand bond; : non-ligand bond; : non-ligand residues involved in hydrophobic contacts; : corresponding atoms involved in hydrophobic contacts. (B) Molecular interactions of linarin inside active site of AChE. Linarin is shown in turquoise blue. Key residues are shown in yellow. AChE is shown in yellow cartoon

The results of AChE inhibitory activity are presented in Figure 1 B. The IC_50_ for the inhibition of AChE by linarin was 3.801 ± 1.149 μM. Molecular docking simulation revealed that linarin was able to penetrate deeply into the catalytic site of AChE despite its large molecule length (Figure 2). The binding mode is shown in Figure 3. 

**Figure 4:**
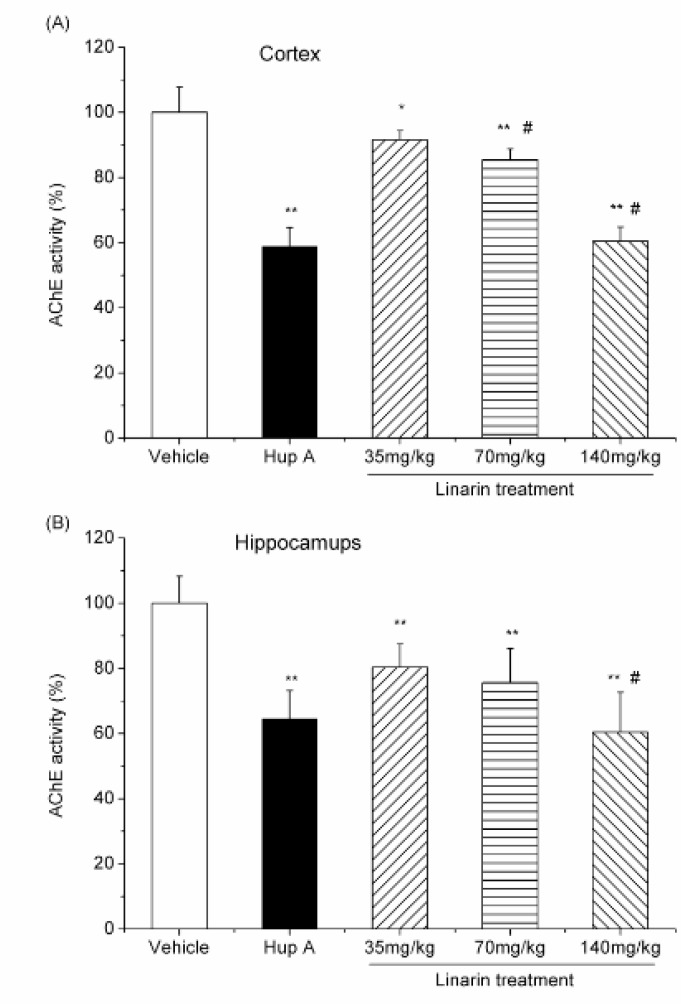
Effect of *i.p*. administration of linarin (35, 70 and 140 mg/Kg) and huperzine A (0.5 mg/Kg *i.p*.) on AChE activity in the mouse cortex (A) and hippocampus (B). Data are expressed as the percentage of control (mean ± SD, *n* = 6). *P < 0.05 vs control; **P < 0.01 vs control; ^#^*P* < 0.05 vs previous dose, Duncan’s test comparison after ANOVA

The inhibitory effects of linarin on AChE activity were also observed* ex-vivo*. After an intraperitoneal administration of linarin with various doses (35, 70 and 140 mg/Kg) to mice, the AChE activity in two brain regions was significantly reduced (*P* < 0.05, Figure 4), with 8.43, 14.4 and 39.5% of inhibition in the cortex and 19.6, 24.4 and 39.6% in the hippocampus (Figure 4).

## Discussion

The AChE inhibitory activity of linarin was evaluated in the concentration range of 0.001–1000 μM. It was found that linarin inhibited the AChE activity in a dose-dependent manner.

Molecular docking simulation revealed that linarin interacted with important amino acid residues surrounding catalytic site through plenty of hydrogen bonding interactions. The 4’-methoxyl group of linarin was in contact with Ser203 through hydrogen bonding, whereas five hydroxyl groups and one oxygen atom of 7-*O*-sugar moiety formed nine hydrogen bonds with Asp74, Asn87, Trp86, Tyr124, Ser125, Tyr337 and His447. The results indicate that 4’-methoxyl group and 7-*O*-sugar moiety may play critical roles in ligand-receptor binding and be essential for AChE inhibition, which are consistent with previous study on structure-activity relationship of flavonoids as AChE inhibitors (15, 16). The hydrogen bonding interactions were further reinforced via hydrophobic contacts of the flavonoid core of linarin with Tyr72, Val73, Gly120, Gly121, Gly122, Gly126, Glu202, Trp236, Phe295, Phe297, Tyr341, Phe338 and Gly448. All of these molecular interactions including hydrogen bonds and hydrophobic contacts of linarin with key residues of AChE could be the possible molecular mechanism for the potent AChE inhibitory activity of linarin.

After an intraperitoneal administration of linarin with various doses, the AChE activity in two brain regions was significantly reduced. Importantly, the inhibition effects produced by high dose of linarin (140 mg/Kg) were the same as that obtained after huperzine A treatment (0.5 mg/Kg) (*P*>0.05). The results indicate that linarin can inhibit AChE activity in both the cortex and hippocampus.

In conclusion, our study demonstrates that linarin exerts a strong AChE inhibitory effect both* in-vitro* and *ex-vivo*. The 4’-methoxyl group and 7-*O*-sugar moiety of linarin may be essential for AChE inhibition. These results help to understand the molecular basis of lianrin for AChE inhibition activity, which may be useful for rational design for anticholinesterasics and the treatment of some diseases associated with AChE in the future.
